# Effect of Foot-and-Mouth Disease Vaccine on Pregnancy Failure in Beef Cows

**DOI:** 10.3389/fvets.2021.761304

**Published:** 2021-11-12

**Authors:** Camila Garcia-Pintos, Franklin Riet-Correa, Alejo Menchaca

**Affiliations:** ^1^Instituto de Reproducción Animal Uruguay, Fundación IRAUy, Montevideo, Uruguay; ^2^Plataforma de Salud Animal, Instituto Nacional de Investigación Agropecuaria, Montevideo, Uruguay; ^3^Programa de Pós-graduação em Ciência Animal nos Trópicos, Escola de Medicina Veterinária e Zootecnia, Universidade Federal da Bahia, Salvador, Brazil

**Keywords:** gestation maintenance, pregnancy losses, embryo mortality, abortion, FMD vaccine, hyperthermia

## Abstract

This study evaluates whether the foot-and-mouth disease (FMD) vaccination increases pregnancy failures in *Bos taurus* beef cows. A total of 3,379 cows were assigned to two experimental groups to receive (*n* = 1,722) or not receive (*n* = 1,657) a FMD vaccine (commercial preparation containing FMD virus, O1 Campos and A24 Cruzeiro) at different gestational age. Pregnancy diagnosis was performed by ultrasonography at vaccination time (Day 0), and the cows were classified by days of pregnancy as follows: (a) <29 days after mating (presumed pregnant cows, *n* = 778), (b) between 30 and 44 days of pregnancy (*n* = 1,100), (c) 45 and 59 days of pregnancy (*n* = 553), and (d) between 60 and 90 days of pregnancy (*n* = 948). Pregnancy failure was determined 30 days after vaccination by a second ultrasound examination. Cows that were vaccinated within 29 days after mating had a 7.8% greater pregnancy failure rate than non-vaccinated cows (44.1%, 163/370 *vs*. 36.3%, 148/408, respectively; *P* <0.05). Cows vaccinated between 30 and 44 days of gestation had a pregnancy failure rate greater than non-vaccinated cows (4.9%, 28/576 *vs*. 2.5%, 13/524, respectively; *P* <0.05). When cows received the vaccine between days 45 and 90 of gestation no differences in pregnancy failure were observed (0.8%, 6/776 *vs*. 1.2%, 9/725, respectively; *P* = NS). Body temperature and local adverse reactions to vaccine inoculation were recorded in a subset of 152 multiparous cows. Hyperthermia (>39.5°C) was detected on Day 1 or 2 in 28.0% (21/75) of vaccinated *vs*. 7.8% (6/77) of non-vaccinated cows (*P* <0.01). Local adverse reaction to the FMD vaccine inoculation increased from 0.0% (0/75) on Day 0, to 15.7% (11/75) on Day 4, and 38.7% (29/75) on Day 10 (*P* <0.01). On Day 30 local reaction was detected in 10.5% (34/323) and fell to 2.2% on Day 60 (7/323) post vaccination (*P* <0.01). In conclusion, FMD vaccine increases pregnancy failure when it is administered before 45 days of gestation, an effect that was associated with hyperthermia and local adverse reaction. No effect on pregnancy failure was found when vaccination was performed after 45 days of gestation.

## Introduction

Foot-and-mouth disease (FMD) is a severe, highly contagious viral disease with devastating economic and social impacts (1, 2). The virus affects cloven hoofed animals such as cattle, sheep, goats, and pigs, and it is listed by the World Organization for Animal Health (OIE) as obligatory declaration with the highest health risk [list A; (3)]. The high virulence, the wide range of hosts, the immunological status of the infected animals, the diversity of the virus variants, the lack of cross-protection among the virus serotype and the high contagious capacity of the FMD virus all explain its presence and re-emergence in various regions of the world (4). Vaccination is an effective measure to control FMD and it has proven to be a highly useful strategy in preventing dissemination and has made elimination of the disease a possibility (5, 6). Routine vaccination is applied to cattle in countries or regions where the disease is endemic, in countries that are recognized as free from FMD as a result of vaccination, as well as in countries where there is a risk of virus entering from neighboring countries or regions.

The economic impact of FMD is not only due to the direct losses caused by emergence of clinical cases, but also for the international trade restrictions on animal products belonging from those countries where the disease is present, or even when the countries are free from the disease because of vaccination (7). In addition, countries that continue vaccinating when the disease is not present as a precaution, still incur indirect costs that are often covered with public expenditure. According to FAO, in countries where routine vaccination is used every year, vaccination represents over 90% of the cost to control the disease (4). Vaccination campaign is one of the main factors determining the vaccine effectiveness, thus, the conviction of farmers to subject their herds to vaccination and to continue implementing rigorous control procedures (4). The knowledge of side effects and safety of vaccination improve with the implementation of official campaigns and allows the establishment of the economic impact of vaccination programs.

Campaigns against FMD try to vaccine the entire bovine population during a given period, which in some countries coincides in time with the breeding period of beef cows. The effect of FMD vaccination on embryonic and/or fetal death losses is not clear and the scientific evidence on this issue is emerging with varying results (8–10). Yet, the concept that the FMD vaccine induces pregnancy losses in cows has been gaining attention among farmers and veterinarians in South America (11, 12). Several factors have hindered clear associations between the FMD vaccine and pregnancy losses such as age of gestation, type of vaccine, nulliparous or multiparous cows, interaction with other diseases, and weaknesses in experimental designs which are often based on field reports. Insufficient information is available to know the effect that FMD vaccine with an oil emulsion compound has when it is given during the breeding season.

The objective of this study was to evaluate the effect of FMD vaccination on pregnancy failure in beef cattle. The vaccine was administered at different gestational ages either in embryonic stages (i.e., within 44 days of pregnancy) or during the fetal stages (i.e., between 45 and 90 days of gestation). The study included the evaluation of pregnancy failure, as well as the incidence of hyperthermia and adverse local reactions after inoculation.

## Materials and Methods

The experiment was conducted at the end of the breeding season, during the period for FMD vaccination established by the official campaign against FMD in Uruguay (i.e., 15th February to 15th March, 2019). All experimental procedures involving animals including injections, temperature measurements, and ovarian and uterine examinations were approved by the Internal Animal Care Committee of *Fundación IRAUy* (protocol number 003/2019) and were conducted in accordance with the guidelines of the National Council of Animal Care (CNEA) of Uruguay.

### Animals and General Management

The study followed 3,379 beef cows, of these 2,331 were nulliparous with body condition score (BCS) 5.0 ± 0.5 [median ± inter-quartile range, 1–8 scale, emaciated to obese; (13)] and 1,048 multiparous cows with BCS 3.5 ± 1.0. The experiment was performed in 10 different locations in 24 replicates. A replicate was considered a group of cows that during the experiment remained together in the same herd, in the same paddock, under the same management conditions, and were subjected to both experimental groups. The animals were maintained grazing on rangeland conditions with unrestricted access to water. As mandated by law in Uruguay, all cows were individually identified soon after birth by electronic chips for traceability, which facilitated data retrieval and processing. One to four weeks before the beginning of the breeding period all the cows received the routine vaccination schedule against bovine viral diarrhea virus 1 and 2, bovine herpesvirus 1 and 5, Campylobacter fetus fetus and *Leptospira spp*.

### Determination of Gestational Age and Experimental Design

**Figure 1:**
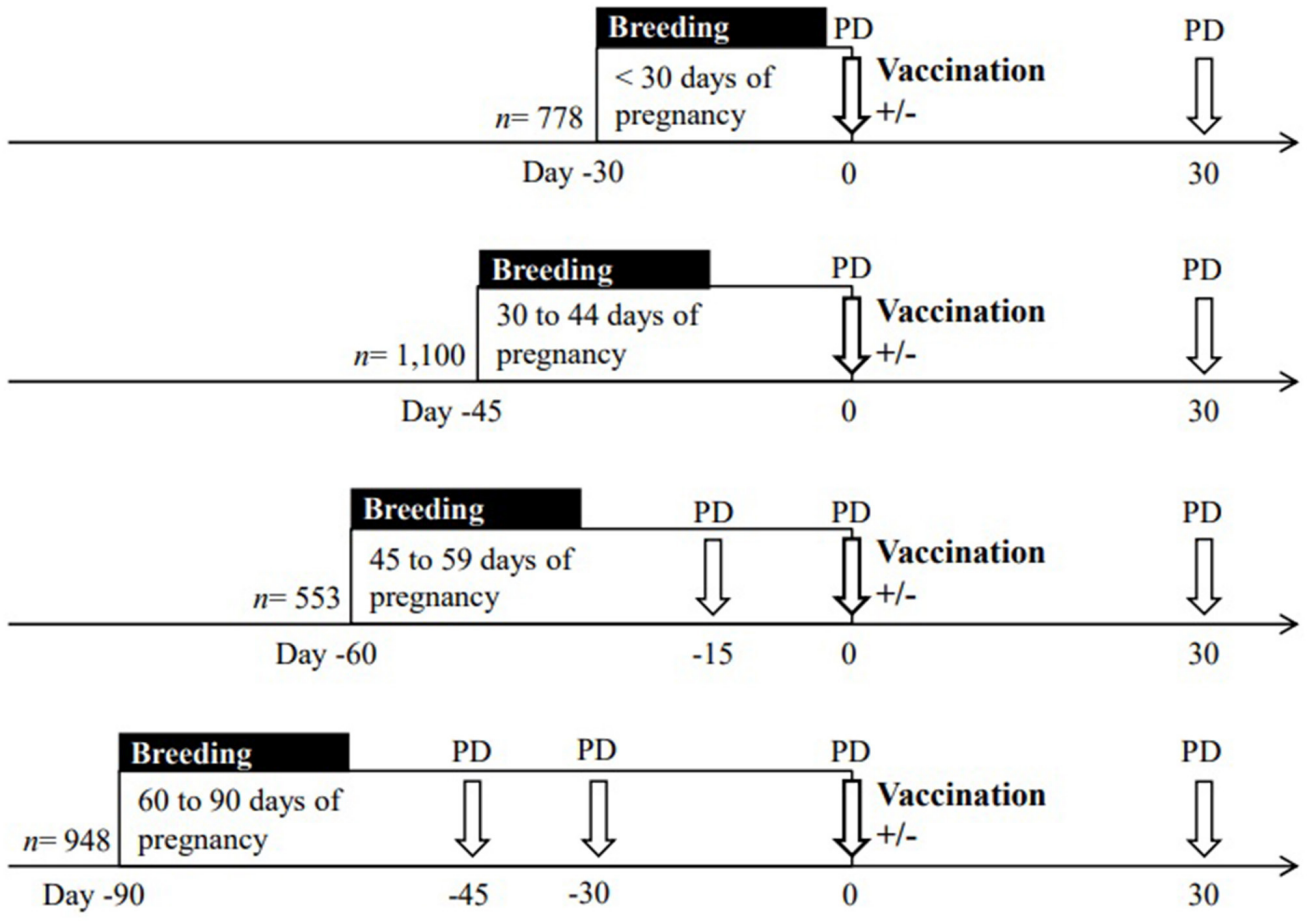
Schematic representation of the experimental design to evaluate the effect of the vaccination (Day 0, *n* = 3,379) against foot-and-mouth disease (FMD) on pregnancy failure in beef cows. For each gestational age, breeding period includes natural service or insemination. The vaccine was administered (+) or not administered (–) at different gestational ages, which were determined by pregnancy diagnosis (PD) performed by ultrasound examination before the beginning of the experiment (i.e., from 30 to 45 days after mating/insemination). For determination of pregnancy failure, PD was repeated in all the cows on Day 0 and on Day 30 post vaccination.

The cows received natural mating or insemination according with the management of each farm. Transrectal ultrasound examinations (5.0 MHz, Sono V6, Shenzhen, China) were performed by the same operator in all cows within 44 d from the beginning of the breeding period. Pregnancy diagnosis and gestational age were determined by the measurement of embryo and fetal crown-rump length in those cows from 30 to 44 d of pregnancy (14). Pregnancy diagnosis was repeated at vaccination time (Day 0 of the experiment), and 1 month later (Day 30) to determine the rate of pregnancy failure (Figure 1). Nulliparous and multiparous cows were classified according to gestational age at the time of vaccination in four categories: (a) cows presumed pregnant, defined by bearing a corpus luteum and had remained with the bulls during the 29 days prior to vaccination (*n* = 778); (b) pregnant cows between 30 and 44 days of gestation (*n* = 1,100); (c) pregnant cows between 45 and 59 days of gestation (*n* = 553); and d) pregnant cows between 60 and 90 days of gestation (*n* = 948). On Day 0, within location and replicates, cows were randomly allocated to two experimental groups to receive or not 2 ml of FMD vaccine (Bioaftogen, series 945, Biogenesis Bagó, Buenos Aires, Argentina). The vaccine consisted of an oil emulsion adjuvant containing FMD virus types O1 Campos and A24 Cruzeiro replicated in BHK suspension cell culture, inactivated with binary ethylenimine and purified with polyethylene glycol as indicated by the manufacturer. The vaccine was administered by subcutaneous injection in the neck region with a 12 mm x 18-gauge needle. Thus, half of each gestational age category was either subjected or not to vaccination, and pregnancy failure was evaluated for both experimental groups. Schematic representation of the experimental design is depicted in Figure 1.

### Determinations

Pregnancy failure was defined as cows that were pregnant on Day 0, or presumed pregnant within 29 days of mating, and were not pregnant on Day 30 determined by ultrasound examination. In addition to the percentage of pregnancy failure for each experimental group, the relative difference between both groups was also calculated, defined as the difference between both experimental groups over the pregnancy failure found in non-vaccinated cows that served as control, as follows: (pregnancy failure of vaccinated – non-vaccinated)/ pregnancy failure of non-vaccinated cows.

### Body Temperature and Local Reaction

Body temperature was determined from Day 0 to Day 4 after vaccination. A subset of 152 pregnant multiparous cows, that corresponded to one replicate, were assigned to receive (*n* = 75) or not (*n* = 77) a dose of FMD vaccine and vaginal temperature was measured once a day (7 am−9 am) by the same operator with digital thermometers (OMRON, Dalián, China). Cows body temperature was dichotomized depending on if cows were hyperthermic or not after vaccination, cows were considered normothermic when the vaginal temperature was <39.5°C, or hyperthermic when vaginal temperature was ≥39.5°C (15). For description of the environmental conditions, humidity and air temperature were recorded, and the temperature-humidity index (THI) was calculated as follows: THI = (0.8 × T°C) + [(RH/100) × (T°C – 14.4)] + 46.4; where T = temperature and RH = relative humidity (16). The THI recorded during the vaginal temperature determinations was 66 on Day 0, 73 on Day 1, 74 on Day 2, 75 on Day 3 and 78 on Day 4.

Local adverse reaction to the vaccination was evaluated by the increase in size of the area at the injection site, which was measured using a caliper for area calculation (height x width) and was classified in small (smaller than 25 cm^2^), medium (between 26 and 100 cm^2^), or large (larger than 100 cm^2^). The fixed cut-off value was 1 cm^2^ and the examination was performed on Days 0, 4, and 10 in a subset of 152 multiparous cows that received (*n* = 75) or did not receive (*n* = 77) a dose of FMD vaccine; and on Days 0, 30, and 60 in a subset of 634 multiparous cows that received (*n* = 323) or did not receive (*n* = 311) vaccine.

### Statistical Analysis

Pregnancy failures for each gestational age (four periods) were compared using generalized linear mixed models (GLMM) with a binomial distribution using multivariate logistic regression by InfoStat software (17). The model included treatment (vaccination *vs*. no vaccination), parity (nulliparous *vs*. multiparous), and their interaction as fixed factors. Location (10 locations), replicate (24 replicates) and individual animal identification (3,379 cows) was included as random factors. Proportion of cows with hyperthermia and with local adverse reaction were analyzed by GLMM with a binomial distribution using multivariate logistic regression, while ANOVA was used for those data showed as continuous variable. The model included treatment (vaccination *vs*. no vaccination), day of the experiment, and their interaction as fixed factors, while individual animal identification was included as random factor. Data showed as continue variables are presented as mean ± SEM. Significance was set at *P* <0.05 with tendencies when 0.05 < *P* <0.10.

## Results

### Pregnancy Failure

Administration of FMD vaccine increased pregnancy failures when it was administered within 29 days after the onset of mating and between days 30 and 44 of gestation (*P* <0.05). No interaction was found between vaccine treatment and parity for any gestational age group (*P* = NS). Statistical model results are presented in Table 1.

**Table 1:** Statistical results of pregnancy failure in beef cows receiving or not receiving vaccination against foot-and-mouth disease (FMD) at different gestational ages.

	**Fixed effects**	***P* value**	**95% CI**	
FMD vaccine administered within 29 days from mating (*n* = 778)				
	Vaccine treatment	0.03	1.04	1.84
FMD vaccine administered from 30 to 44 days of gestation (*n* = 1,100)
	Vaccine treatment	0.04	1.03	3.92
	Parity	0.10	0.30	1.10
	Treatment x parity	0.79	−0.06	0.05
FMD vaccine administered from 45 to 59 days of gestation (*n* = 553)				
	Vaccine treatment	0.64	−0.02	0.01
	Parity	0.76	0.02	1.11
	Treatment x parity	0.99	−0.04	0.03
FMD vaccine administered from 60 to 90 days of gestation (*n* = 948)				
	Vaccine treatment	0.88	0.13	2.35
	Parity	0.99	−0.03	0.01
	Treatment x parity	0.99	−0.04	0.05

*Treatment (vaccination vs. no vaccination) and parity (nulliparous vs. multiparous) were included as fixed factors in the statistical model. CI, confidence intervals*.

Pregnancy failure in cycling cows presumed pregnant that received vaccination within 29 days after the onset of mating were affected by the treatment (*P* <0.05). A larger percentage of cows were not pregnant 30 days after FMD vaccine administration (44.1%, 163/370) compared to those cows that were not vaccinated (36.3%, 148/408; *P* <0.05). Pregnancy failure was 7.8% higher for vaccinated cows, and the relative difference was 21.5% higher compared with non-vaccinated cows (Table 2).

**Table 2:** Pregnancy failure in beef cows after receiving a vaccination against foot-and-mouth disease (FMD) at different gestational ages.

	** <29 days of pregnancy (*n* = 778)**	**30–44 days of pregnancy (*n* = 1,100)**	**45–59 days of pregnancy (*n* = 553)**	**60–90 days of pregnancy (*n* = 948)**
With FMD vaccine	44.1% (163/370)	4.9% (28/576)	1.0% (3/286)	0.6% (3/490)
Without FMD vaccine	36.3% (148/408)	2.5% (13/524)	1.5% (4/267)	1.1% (5/458)
*P* value	<0.05	<0.05	NS	NS
Difference^a^	7.8%	2.4%	ND	ND
Relative difference*^b^*	21.5%	96.0%	ND	ND

a *Difference is shown when P <0.05 (ND: Not determined)*.

b *Relative difference in pregnancy failure is the difference between both experimental groups over the pregnancy failure of non-vaccinated cows that served as controls*.

The pregnancy failure rate was greater in those cows that received vaccination between 30 and 44 days of gestation compared with non-vaccinated cows (4.9%, 28/576 *vs*. 2.5%, 13/524, respectively; *P* <0.05). There was no difference in the rate of pregnancy failure in vaccinated and non-vaccinated cows that at the time of vaccination were between 45 and 59 days of gestation (1.0%, 3/286, *vs*. 1.5%, 4/267, respectively), and between 60 and 90 days of gestation (0.6%, 3/490, *vs*. 1.1%, 5/458, respectively) (*P* = NS). The results are shown in Table 2.

Pregnancy losses were greater in the embryonic stages (between 30 and 45 days of gestation) than in fetal stages (between 45 and 90 days of gestation), 3.7% (41/1,100) *vs*. 1.0% (15/1,501), respectively (*P* <0.05).

No interaction was found for pregnancy failure between vaccination/non-vaccination and nulliparous/multiparous cows (*P* = NS). Regardless of vaccine treatment, the pregnancy failure rate from 30 to 60 days of gestation tended to be greater in nulliparous (4.0%, 21/527) than in multiparous cows (2.4%, 27/1,126) (*P* = 0.07), whereas no differences were found between nulliparous and multiparous that were between 61 to 90 days of gestation (0.9%, 8/847 *vs*. 0.0%, 0/74, respectively; *P* = NS).

### Body Temperature

Body temperature measured intravaginally consistently increased after FMD vaccination. Significant differences between vaccinated and non-vaccinated cows were detected on Day 1 (39.0 ± 0.1 *vs*. 38.6 ± 0.1°C, respectively; *P* <0.01) and on Day 2 (39.0 ± 0.1 *vs*. 38.8 ± 0.1 °C, respectively; *P* <0.01). While 28.0% (21/75) of the animals that received the vaccine reached ≥ 39.5°C (hyperthermia) on Days 1 or 2 after vaccination, 7.8% (6/77) of the non-vaccinated cows reached this temperature (*P* <0.01). Only five cows that received vaccine had hyperthermia for more than 24 h (*i.e*., in two consecutive determinations on Day 1 and 2). The results are presented in Table 3.

**Table 3:** Percentage of cows with hyperthermia (≥ 39.5°C) after receiving or not vaccination (Day 0) against foot-and-mouth disease (FMD).

	**Day 0**	**Day 1**	**Day 2**	**Day 3**	**Day 4**
With FMD vaccine (*n* = 75)	0.0%^a^	14.7%^b^	20.0%^b^	0.0%^a^	0.0%^a^
	(0/75)	(11/75)	(15/75)	(0/75)	(0/75)
Without FMD vaccine (*n* = 77)	0.0%^a^	1.3%^a^	7.8%^a^	0.0%^a^	0.0%^a^
	(0/77)	(1/77)	(6/77)	(0/77)	(0/77)

*Different superscripts between treatments show significant differences (a vs. b, P <0.05)*.

### Local Adverse Reaction to FMD Vaccine

The proportion of cows that had local adverse reaction at the injection site increased from Day 0 (0.0%) to Day 10 post vaccination (38.7%, *P* <0.01). In those cows that local adverse reaction was evaluated on Days 0, 30 and 60 post vaccination, the proportion of animals that had a local reaction increased from 0.0% to 10.5% on Day 30, and then fell to 2.2% on Day 60 of the experiment (*P* <0.01; Table 4). There was no difference in the proportion of cows that had different sizes of local reaction on Day 4 that was 8.0% (6/75), 4.0% (3/75) and 2.7% (2/75), for small, medium and large reactions, respectively (*P* = NS). On Day 10 there were more animals with small (18.7%, 14/29) than large reaction (6.7%, 5/75, *P* <0.05), whereas the percentage of animals that had medium local reaction was intermediate (13.3%, 10/75; *P* = NS). Non-vaccinated cows that were evaluated at the same time as vaccinated cows did not show any kind of reaction in the neck region.

**Table 4:** Percentage of cows with local adverse reactions defined as the increase in size of the area at the injection site within 4 days after the vaccination (Day 0) against foot-and-mouth disease administered in the neck region by subcutaneous injection.

	**Day 0–10**			**Day 0–60**		
	**Day 0**	**Day 4**	**Day 10**	**Day 0**	**Day 30**	**Day 60**
Local reactions	0.0%^a^ (0/75)	14.7%^b^ (11/75)	38.7%^c^ (29/75)	0.0%^a^ (0/323)	10.5%^b^ (34/323)	2.2%^c^ (7/323)

*Different superscripts represent significant differences (P <0.05)*.

*Two subsets of cows were evaluated from Day 0–10 (n = 152) or from Day 0–60 after vaccination (n = 634). No cows in non-vaccinated group had local reaction in the neck region*.

## Discussion

This study shows that the FMD vaccine increased pregnancy failure in beef cows when it was administered within 44 days of gestation, while no effect was observed when the vaccine was administered beyond 45 days of pregnancy. Vaccination was associated with hyperthermia that occurred during the next 2 days that followed inoculation and produced local reactions in the injection site that in some cases persisted for 1 month.

Vaccine administration within a month after mating increased pregnancy failure, since the percentage of cows that failed to be pregnant was greater in vaccinated than in non-vaccinated cows. This difference was 21.5% among vaccinated and non-vaccinated cows. The pregnancy failure rate increased 96.0% in vaccinated cows compared with non-vaccinated cows (4.9 and 2.5%, respectively) when the inoculation was performed between days 30 and 44 of gestation. Based on this finding, the effect of vaccination on the establishment and maintenance of pregnancy ought to be further researched. Hansen et al. (18) reported that immunogenicity after the administration of a vaccine can compromise female fertility and interfere with establishment of pregnancy. Interactions between components of the maternal immune system and the *conceptus* can be either beneficial or harmful to pregnancy establishment and maintenance (19–22). Studies in cattle suggest that infectious disease outside the reproductive tract can lead to a reduced pregnancy rate (21). There are two different hypothetic mechanisms by which vaccination could negatively affect fertility. One of them is hyperthermia, which in this study occurred in the 28% of the vaccinated animals, which may block embryonic development or inhibit secretion of luteinizing hormone and progesterone. Hyperthermia causes a redistribution of blood flow away from the reproductive tract to the periphery and can cause decreased corpus luteum activity (18). Progesterone clearance may also be promoted during hyperthermia (23), a process that can cause embryonic death and pregnancy failure (24), which was found in a proportion of cows that were within 44 d of pregnancy (i.e., during the embryonic stage of pregnancy). Another hypothesis is that the inflammatory cascade induced by vaccination can impair embryo development or luteal function, resulting in pregnancy failure. Proinflammatory cytokines and prostaglandin interfere with luteal maintenance and embryonic development (18). Further studies are needed to determine the mechanism by which FMD vaccination increases pregnancy failures when it is administered during embryonic stages of gestation (within 44 days of pregnancy) in beef cows. Moreover, although there was a much greater risk (96.0%) of pregnancy failure when FMD vaccine was administered between days 30 and 44 of gestation than when it was administered within 29 days of gestation (21.5%). The increase was only 2.4 percentile points on cows vaccinated between days 30 and 44, and 7.8 percentile points on cows vaccinated within 29 days of gestation.

The finding that FMD vaccination increases pregnancy losses when it is administered between days 30 and 44 of gestation in *Bos taurus* cows supports similar results reported in *Bos indicus* by Ferreira *et al*. (8). In that study, the administration of FMD vaccine (5 ml via subcutaneous, Ourovac Aftosa, Ourofino Saúde Animal, Cravinhos, SP, Brazil) on day 31 of gestation stimulated an acute-phase protein reaction causing an increase in body temperature, and an increase in pregnancy losses that was 3.9% (7/180) *vs*. 16.5% (29/176) for non-vaccinated and vaccinated cows, respectively (*P* <0.05). Interestingly, the effect of vaccination on pregnancy failure was greater than that found in the current study. However, the conditions were different, *e.g*., different adjuvant, different vaccine, different doses, different breed of cows, among others. Vaccines formulated to control FMD content inactivated antigens that are poorly immunogenic, thus, selected adjuvants are required to increase their immunogenicity and extend the duration of protection (25). Various adjuvants can stimulate the synthesis of proinflammatory cytokines of different intensity (26) inducing different levels of local and systemic inflammatory responses (27), with consequences for the expected reaction (28). The evaluated vaccine in the study reported by Ferreira *et al*. contained an oil-based adjuvant associated with saponin, a substance that is not present in the vaccine used in the current study (based on an oil emulsion adjuvant). Saponin can stimulate cellular immunity response more strongly than mineral oil-based adjuvants (29), and probably could explain, at least in part, the differences between both studies. Furthermore, Marqués *et al*. (9) did not find significant difference in pregnancy failure among cows that received or not receive the FMD vaccine on day 33 of gestation, 3.7% (6/162) *vs*. 1.3%, (2/149) for animals with and without vaccine, respectively (*P* = NS). The aforesaid study was performed with the same vaccine formulation as used in the current study. Although the authors reported no significant difference between experimental groups, they suggested to repeat the study with a greater number of females to obtain a clear conclusion. The current study adds further information with a total of 1,878 cows within 44 days of gestation and shows that FMD vaccination has a negative effect on the establishment or maintenance of pregnancy.

The FMD vaccine used in the current study did not affect pregnancy maintenance when administered after Day 45 of gestation. Gestational age often is classified in embryonic stage (earlier than 42–45 days) and fetal stage (from 42 to 45 days of pregnancy) (30, 31). Fetal stage is characterized by rapid fetal growth, maturation of organs and systems (32), and during this period pregnancy is firmly established and pregnancy losses are reduced (33, 34). The embryonic stage is more critical in terms of pregnancy maintenance, since several events of crucial importance for the future of the embryo occur during this period (35, 36). In the current study, regardless of FMD vaccine treatment, pregnancy failure in both vaccinated and non-vaccinated cows was greater before 44 days of gestation than afterwards. According to a recently published meta-analysis done on beef cows (37), 48% of the embryos are lost during the early embryonic period of gestation (*i.e*., before day 32), and 6% are the losses between days 29 and 100 of gestation. The fact that pregnancy maintenance is more labile during the embryo stage may explain why the FMD vaccine induced pregnancy failure within 44 days of pregnancy and no effect was observed when it was administered during the fetal stages.

Vaccination against FMD induced hyperthermia and adverse reaction in the inoculation site. The immune system is a network of specialized cell types and tissues that communicate via cytokines and direct contact, to orchestrate specific types of defensive responses (38). After vaccination, dendritic cells interact with specialized pattern recognition receptors to activate the innate immune system (39, 40). Macrophages and dendritic cells secrete proinflammatory cytokines that recruit and/or activate antigen presenting cells at the inflammation site, enhancing antigen presentation capacity and migrating to lymphoid tissues where the dendritic cells interact with T cells and B cells to initiate and develop the adaptive immune response (41). Different vaccine adjuvants have different types of activation of dendritic cells and induce different levels of local site inflammation (40). Indeed, inflammation is a key part of the activation of innate immune system after vaccination. The adverse local reaction observed in the present study may be associated with the local overproduction of cytokines, generated by the adjuvant present in the vaccine. Local inflammation induced by the vaccine inoculation likely exacerbated release of proinflammatory cytokines that reach the hypothalamus and other cerebral branches, stimulating the production of prostaglandin-E2, which induces hyperthermia (39, 40). In the current study, the proportion of vaccinated animals that had hyperthermia and local reaction decreased as days went on. Regarding hyperthermia, all the cows were normothermic by day three after vaccination. The local adverse reaction at the injection site persisted for a longer time, and 60 days post vaccination only 2% of the vaccinated cows showed a local reaction.

The FMD is one of the most economically significant livestock diseases (7), and the economic impact of this level of pregnancy failure, surely support the beneficial of vaccination compared to a no vaccination FMD control strategy (42). The results of this study lead to a clear conclusion of the effect of FMD vaccine on gestation maintenance in cattle and has important implications for the official control programs in countries where FMD vaccination is mandatory.

## Conclusion

The FMD vaccine increases pregnancy failure when it is administered before 45 days of pregnancy in *Bos taurus* beef cows. Pregnancy failure was associated with hyperthermia and local adverse reaction in the vaccine inoculation site in many cases. When the FMD vaccine is administered after 45 days of gestation (*i.e*., during the fetal stage of pregnancy) pregnancy maintenance was not affected. This finding suggests the convenience to administer the FMD vaccine after 45 days of the end of the breeding season in beef cattle, which have practical implications in the fine-tuning of the official campaigns against this devastating disease.

## Data Availability Statement

The original contributions presented in the study are included in the article/supplementary material, further inquiries can be directed to the corresponding authors.

## Ethics Statement

The animal study was approved by Internal Animal Care Committee of Fundación IRAUy.

## Author Contributions

All authors conceived and designed the study. CG-P was in charge of the field trials. CG-P and AM did data analysis and interpretation, as well as wrote the manuscript. All authors have read and approved the final manuscript.

## Conflict of Interest

The authors declare that the research was conducted in the absence of any commercial or financial relationships that could be construed as a potential conflict of interest.

## Publisher's Note

All claims expressed in this article are solely those of the authors and do not necessarily represent those of their affiliated organizations, or those of the publisher, the editors and the reviewers. Any product that may be evaluated in this article, or claim that may be made by its manufacturer, is not guaranteed or endorsed by the publisher.
